# A Survey of Bioinspired Jumping Robot: Takeoff, Air Posture Adjustment, and Landing Buffer

**DOI:** 10.1155/2017/4780160

**Published:** 2017-09-14

**Authors:** ZiQiang Zhang, Jing Zhao, HanLong Chen, DianSheng Chen

**Affiliations:** ^1^College of Mechanical Engineering and Applied Electronics Technology, Beijing University of Technology, Beijing 100124, China; ^2^School of Mechanical Engineering and Automation, Beihang University, Beijing 100191, China

## Abstract

A bioinspired jumping robot has a strong ability to overcome obstacles. It can be applied to the occasion with complex and changeable environment, such as detection of planet surface, postdisaster relief, and military reconnaissance. So the bioinspired jumping robot has broad application prospect. The jumping process of the robot can be divided into three stages: takeoff, air posture adjustment, and landing buffer. The motivation of this review is to investigate the research results of the most published bioinspired jumping robots for these three stages. Then, the movement performance of the bioinspired jumping robots is analyzed and compared quantitatively. Then, the limitation of the research on bioinspired jumping robots is discussed, such as the research on the mechanism of biological motion is not thorough enough, the research method about structural design, material applications, and control are still traditional, and energy utilization is low, which make the robots far from practical applications. Finally, the development trend is summarized. This review provides a reference for further research of bioinspired jumping robots.

## 1. Introduction

A jumping robot can cross the obstacle several times its own height and has a good ability to avoid risks [1]. For example, the flea-inspired jumping designed by Noh et al. can jump a distance of up to 30 times its body size [2], and miniature a jumping robot designed by Kovac et al. can jump obstacles more than 27 times its own size [3]. The strong jumping ability of this type of robots makes it possible to move in a complex and changeable environment with big obstacles, and it has potential application value in many fields, such as star detection, disaster rescue, and military reconnaissance. This greatly widens the application field of robots [4, 5].

According to different structure forms, the jumping robot can be divided into two types: nonbionic jumping robot and bioinspired jumping robot. The nonbionic jumping robot is a type of robot which does not have the shape characteristics or movement characteristics of a creature and only is designed according to the actual needs. For example, internal combustion driving is used for some nonbionic jumping robot, which can jump by kinetic energy of the jumping robot converted by the high temperature and high-pressure gas generated by the combustion of the combustible mixture gas doing the work [6–8], and this drive method is completely different from the creatures. In nature, the creature has a good adaptability to the environment after a long period of evolution, and it shows a high rationality in the physiological structure, motion control, and posture adjustment [9–12]. Therefore, it is one of the important research directions to develop a robot system that can simulate the structure and function of creatures to expand the motion function of the traditional robots. Bioinspired jumping robots are designed using the bionic ideas on the basis of revealing the jumping movement mechanism of creature with jumping ability. It can simulate the efficient and stable jump process of creature and has high jumping ability [13].

In the field of bioinspired jumping robots, most of the early research focused on simulating the jumping process of large animals, such as kangaroo [14]. With the development of bionics, material science, biomechanics, and control science, the latest research results of various related disciplines are gradually applied to the study of bioinspired jumping robot, so that the bioinspired jumping robot develops from imitating the macroscopic movement to the miniaturization and integration of material and structure [4].

The jumping process of creatures or bioinspired jumping robots can be divided into three stages, namely, takeoff stage, air posture adjustment stage, and landing buffering stage. The takeoff process can determine the takeoff speed, jumping height, and jumping distance and then determine the obstacle performance. In air posture adjustment stage, the creatures or robots should be able to control the body posture in the air to achieve a stable motion state, and it also provides the basis for a good landing [15]. Because the landing speed is large (e.g., the landing velocity of the bioinspired jumping robot designed by Zhang et al., which is approximately equal to takeoff velocity without other influence, is about 4.4 m/s [16]), if there is no good landing buffering mechanism, the creatures are prone to overturning or rollover. So the landing buffering stage determines whether the creatures or robots can continue the next movement [17]. These three stages are important to the jumping process, and the bioinspired jumping robot should be able to maintain good movement performance in above three stages, so as to achieve good jumping ability.

This paper summarizes the research status of the bioinspired jumping robots for takeoff stage, air posture adjustment stage, and landing buffering stage, and the movement performances of the bioinspired jumping robots are analyzed and compared. On this basis, the limitations and future development trends are analyzed.

## 2. Research Status of Bioinspired Jumping Robot

### 2.1. Takeoff Stage

The creature with jumping ability can achieve steady and efficient takeoff, and the study of takeoff mechanism of creature is the basis of the design of bioinspired jumping robot. The articular structure [18], muscle movement mechanism [19], movement pattern [20], and energy conversion [21, 22] of many mammals and insects with jumping ability, such as kangaroos [23], locusts [24–26], crickets [27], fleas [28], and froghopper [29], are studied, and the design of the structure and motion pattern of the bioinspired jumping robot mostly draws on the takeoff movement mechanism of the creature to achieve good jumping performance. According to the different driving modes, the bioinspired jumping robot can be divided into three types: pneumatic drive, spring drive, and flexible material drive.

#### 2.1.1. Pneumatic Drive

**Figure 1:**
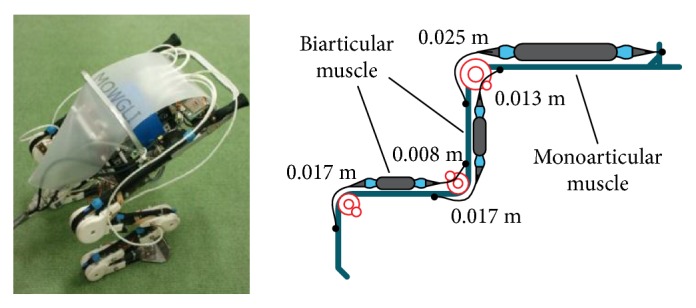
Jumping robot Mowgli [30].

Pneumatic drive has the advantage of good compliance, large driving force, and generally powerful and highly dynamic. *Mowgli* is a typical pneumatic drive jumping robot designed by Niiyama et al. of University of Tokyo (Figure 1). The bionic design principle is used for the leg structure. Each leg consists of hip, knee, and ankle joints. Considering the musculoskeletal system of animals can make them have the ability to move in a huge variety of environments, the artificial musculoskeletal system is proposed based on the engineering concept of using biological structures. It consists of six McKibben pneumatic muscle actuators, which has the characteristic of similarity in length-load curves between the pneumatic muscle and biological muscle. The length of *Mowgli* is 0.9 m, and the weight is only 3 kg. The maximum jump height is about 0.5 m, which is more than 50% of its body height [30].

**Figure 2:**
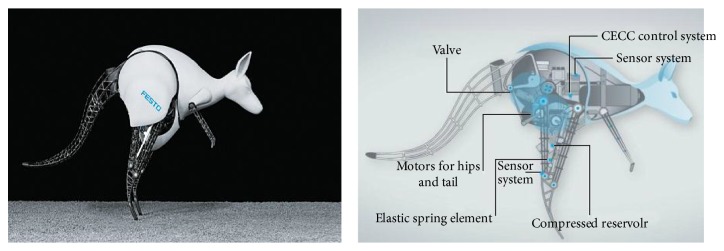
Kangaroo jumping robot designed by FESTO [31].

In 2013, FESTO developed a kangaroo jumping robot (Figure 2). A FESTO DSNUP 20 pneumatic lightweight cylinder is attached along each leg, which actuates the legs. The knee joint and ankle joint are connected via a so-called positive kinematic device, resulting in an interlinked movement sequence. The motors are also used to achieve other auxiliary movement. The weight of the kangaroo jumping robot is 7 kg, the jump distance is 80 cm, and the jump height is 40 cm, which is about 0.7 times its body size [31]. The experimental results of *Mowgli* and the kangaroo jumping robot show that the robot can achieve steady jumps with the pneumatic drive.

#### 2.1.2. Spring Drive

The spring has the advantages of strong energy storage, fast energy release, simple structure, and simple control. So it has been widely used in the design of bioinspired jumping robots to replace the biological leg muscles to achieve energy storage and release.

**Figure 3:**
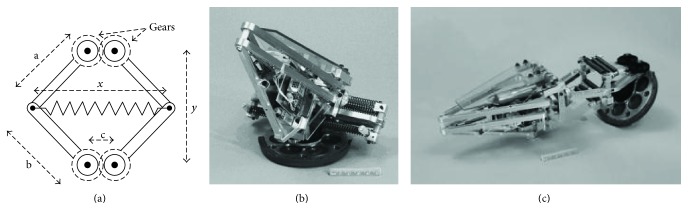
Jumping robot designed by NASA. (a) Schematic diagram of a 6-bar geared mechanism; (b) jumping robot in compressed state; (c) jumping robot in uncompressed state [32].

As early as 1880s, the National American Aeronautics and Space Administration (NASA) developed a jump robot by imitating the jumping process of the frog. To solve the problems of inefficiency and high holding force, a combined 6-bar geared mechanism with spring being used was designed (Figure 3(a)). The spring is linear, and the 6-bar geared mechanism can be considered as a nonlinear spring. The robot can jump through an instantaneous contraction of the spring. The weight of the robot is about 1.3 kg, the jumping height can reach 80 cm, and the horizontal distance is 1.8 m. It can complete all the typical action process of intermittent jump and show high energy conversion efficiency and jumping ability. The compressed state and uncompressed state of the jumping robot are shown in Figures 3(a) and 3(b), respectively [32, 33].

**Figure 4:**
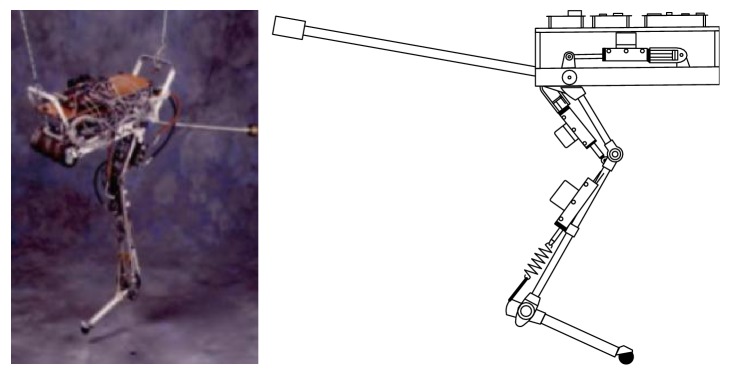
Jumping robot Uniroo [14, 35].

The legs of the creature include the hip, knee, and ankle joints, which are typical series mechanism. The leg structures of many bioinspired jumping robots mimic the biological leg structure and have good jump performance [34]. In 1991, Zeglin et al. designed a one-legged jumping robot Uniroo, which mimics kangaroo locomotion in order to gain insight into the nature of jumping (Figure 4). There are three spatial DOF (degree of freedom) and four actuated DOF (tail, hip, knee, and ankle joints). In the compression phase, the vertical energy is stored in the ankle spring, and in the thrust phase, the vertical energy is recovered from the spring. The experiments show that the robot can achieve a smooth jump [14, 35].

**Figure 5:**
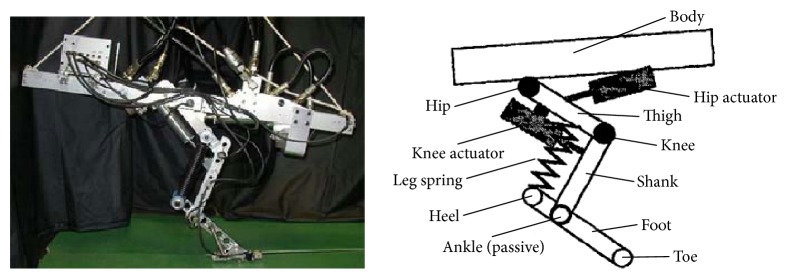
Jumping robot *KenKen* [36].

Hyon et al. of the Department of Mechatronics and Precision Engineering of Tohoku University designed a one-legged jumping robot *KenKen* by imitating the hindlimb of a dog (Figure 5). The leg is made up of the hip, knee, and ankle joints, which is the same as the physiological structure of a biological leg. It has two active joints, hip and knee, and one passive joint, ankle, and there is no actuator at foot. The most distinctive feature of this model is the arrangement of the leg spring. The leg spring is attached between thigh and heel parallel to the shank-like gastrocnemius or plantaris. The arrangement of the leg spring in this way can not only absorb energy in the stance phase of robot but also translate into the kinetic energy of the next jump cycle. The weight is 13.26 kg, and the jumping height is about 0.55 m. The jumping period of *KenKen* is about 0.5 s, and the pitch angle is oscillating within 0° to 15°, which means that the robot is stable [36, 37].

**Figure 6:**
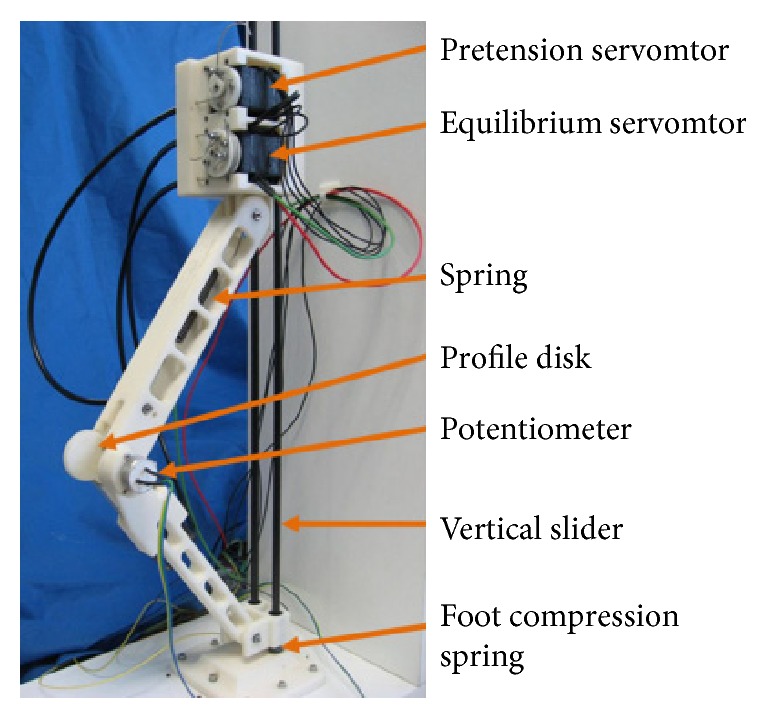
Jumping robot *Chobino1D* [38].

In 2011, Vanderborght et al. designed a jumping robot *Chobino1D* (Figure 6), and a novel version of the MACCEPA (Mechanically Adjustable Compliance and Controllable Equilibrium Position Actuator) actuator is proposed, which can improve the jumping performance and mitigate the impact. For the jumping robot *Chobino1D*, the spring is still the main drive for jumping [38].

For the jumping robots *Uniroo*, *KenKen*, and *Chobino1D*, whose leg structure and biological leg structure are almost the same, the springs approximately simulate the leg muscles to achieve the storage and release of energy. The above studies also show that the spring can drive the *bioinspired* jumping robots with large mass to jump. However, the jump height/body height ratio is not too high. With the broadening of the robot application fields, the jumping robots are gradually expected to be applied in some fields with rugged environments, such as postdisaster rescue and military reconnaissance [13], and these fields require the bioinspired robots to have good concealment and ability to move in a narrow environment. So the bioinspired jumping robot gradually develops toward the miniaturization. Robots are no longer confined to simulating the jumping process of large creatures but are gradually starting to simulate the jumping movement of insects. With the development of manufacturing technology, many miniature bioinspired jumping robots have been developed. Because the size of the spring can be small, it can also be used to drive a micro jumping robot.

**Figure 7:**
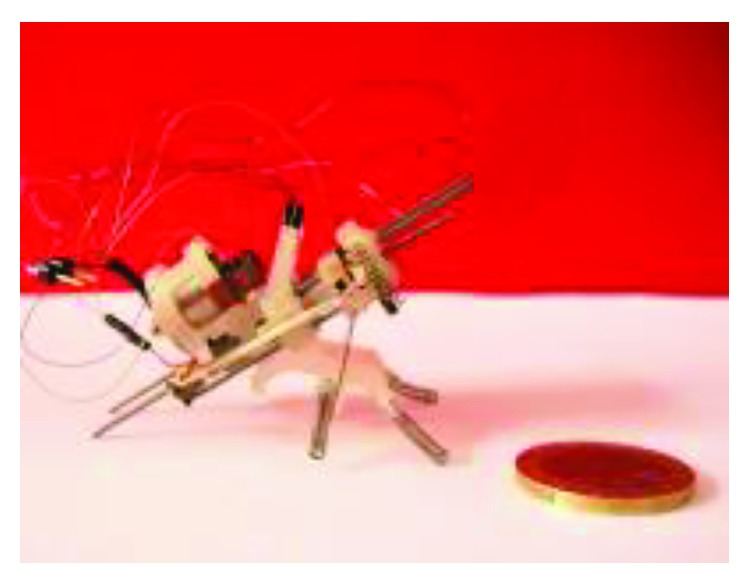
Jumping robot Grillo [39].

**Figure 8:**
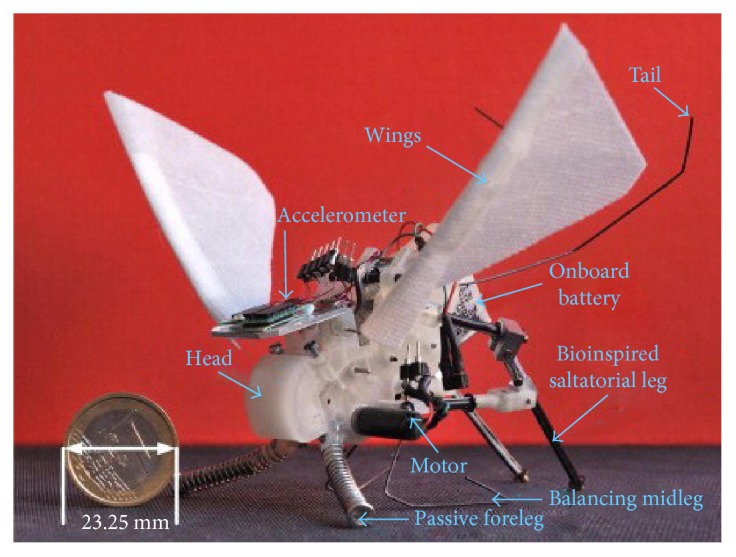
Jumping robot Grillo III [43].

Scarfogliero and Li have developed a multigeneration micro jumping robots which are driven by springs. In 2006, they designed a bioinspired jumping robot Grillo (Figure 7). Inspired by nature, the actuation of the proposed robot is entrusted to loaded springs. During the flight phase, energy from an electric micromotor is collected in springs, while it is released by a click mechanism during takeoff. The robot Grillo is 20 mm long and weighs about 20 grams. Experiments show good performances, with length of the jump equal to 5 body lengths [39–41]. By improvements on the original prototype (Grillo II was designed in 2009 as the basis for a new prototype [42]), in 2012, they designed a new generation of biomimetic inspiration jumping robot Grillo III (Figure 8). Thre saltatorial leg of Grillo III mimics the leg structure of insects, which includes femur and tibia. A natural muscle-tendon system is designed as a spring-segmental gear system to transmit continuous rotation of a DC motor to a reciprocal load-release motion so as to generate continuous jumps. The jumping height of Grillo III is about 200 mm. Compared with Grillo, the jumping height and the stability of the movement have been improved [43].

**Figure 9:**
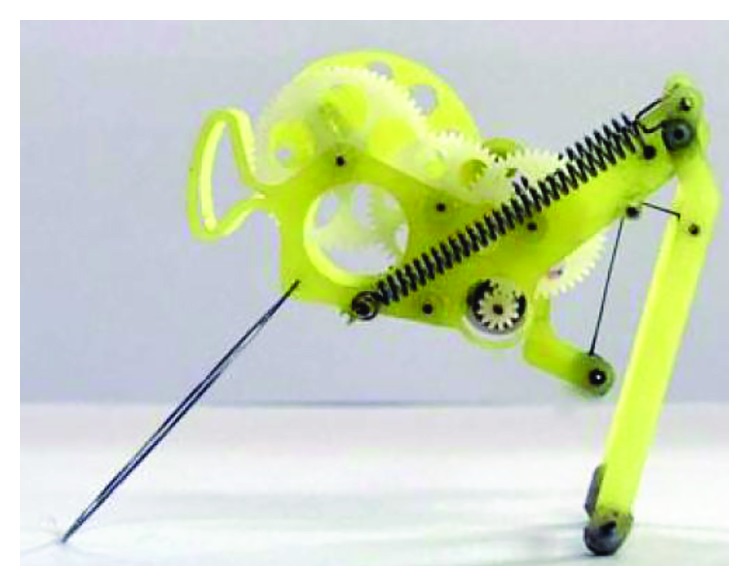
Locust-like jumping robot [44].

In 2012, Nguyen et al. designed a locust-like jumping mechanism for small-scale robots (Figure 9). The shape of the tibia is similar to the real locust leg, and it can rotate about its pivot. A spring as a passive extensor muscle and its tendon are used to achieve the release of energy. One end of the spring is attached to the tibia on one side of the pivot while the other end of the spring is attached to the body frame. The robot can achieve rapid takeoff with the drive of an incomplete cam. This locust-like jumping robot can jump over obstacles of about 14 times and a jumping distance of about 20 times its own size [44]. Compared with Grillo and Grillo III, it shows stronger jumping ability.

**Figure 10:**
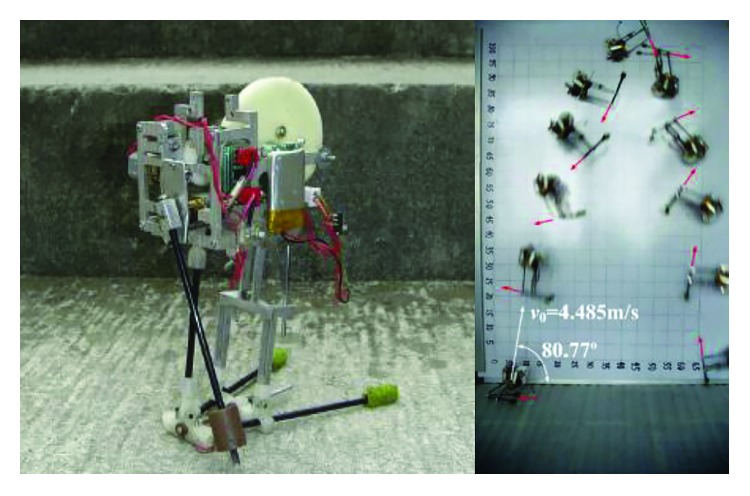
Micro jumping robot [45].

The linear spring can approximately simulate the leg muscles. In order to further reduce the size of the structure, the torsional springs are used in some joints of jumping robots. Zhang et al. of Southeast University presented a jumping robot inspired by jumping locomotion of locusts (Figure 10). Different from a locust-like jumping robot shown in Figure 9, the jumping leg is a four-bar mechanism and has only one DOF. The torsion springs are used to store energy, and the quick release of the torsion springs is realized by the eccentric cam driven by the motor to make the elastic potential energy translated into the kinetic energy of the robot. A pole leg is used to implement the function of takeoff angle adjusting before takeoff and self-righting when the robot is overturned or rollover after landing. Experimental results show that the constructed robot can jump more than 88 cm high at a takeoff angle of 80.33° [45].

**Figure 11:**
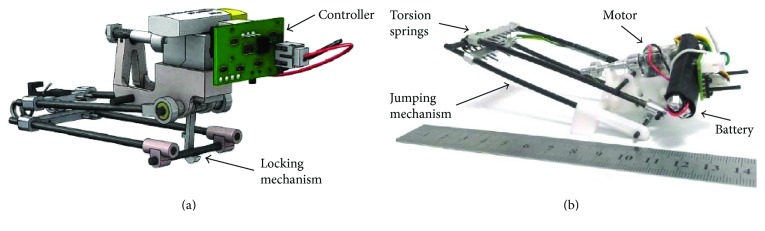
Autonomous 23 g miniature jumping robot. (a) 3D model; (b) prototype [46].

In 2015, Zaitsev et al. also designed a locust-inspired miniature jumping robot. This jumping robot utilizes a pair of legs, each with two equal segments (femur and tibia), inspired by the hindleg of the locust. Torsion springs, as the elastic elements, are located in the joints between the two segments of each leg and connect them. The loaded position and unloaded position of the robot are shown in Figure 11. The locking mechanism is used to realize the quick release of the torsion spring. The most advanced jumping robot demonstrator is power autonomous, weighs 23 g, and is capable of jumping to a height of 3.35 m, covering a distance of 1.37 m [46]. Compared to the other two locust-like jumping robots [44, 45], the jumping ability of the robot which is designed by Zaitsev et al. has been greatly improved.

**Figure 12:**
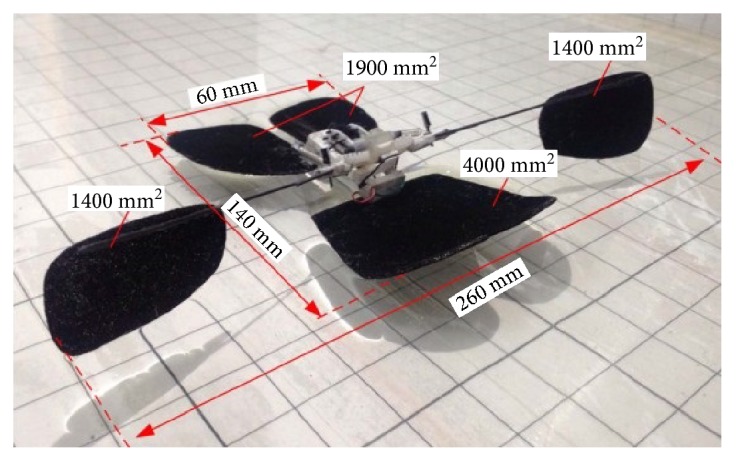
Fabricated water-jumping robot [47].

In order to further extend the environmental adaptability of the robot, the research object of the bioinspired jumping robot is not only limited to the creatures that can jump on the land but gradually imitates the creatures that can jump on the water. Yang et al. of State Key Laboratory of Robotics and Systems of Harbin Institute of Technology designed a water-walking robot by mimicking the jumping abilities of water striders (Figure 12). A spring-based actuating mechanism is proposed to produce a large jumping force. The center of gravity of the robot is carefully designed to allow the robot to jump on the surface continuously and smoothly. The fabricated robot weighs approximately 10.2 g and can continuously jump on water with a maximum leap height and length of 120 and 410 mm, respectively [47].

#### 2.1.3. Flexible Material Drive

**Figure 13:**
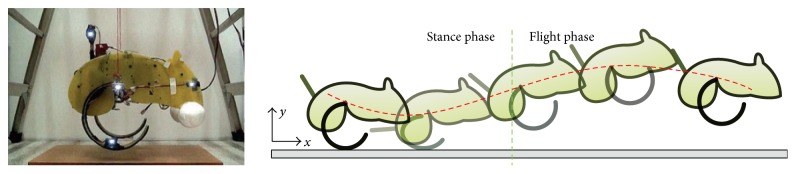
Bioinspired jumping kangaroo robot designed by National Taiwan University [48].

With the development of material science, new materials are gradually applied to the bioinspired jumping robot, which makes the robot with lighter weight and simpler structure. Liu et al. in National Taiwan University designed a bioinspired jumping kangaroo robot, and the half-circular leg is used for this robot (Figure 13). The material of the leg is fiberglass. During the stance phase, the half-circular leg is compressed and stores the potential energy, functioning like the tendon of the kangaroo. When the half-circular leg recovers back, the stored potential energy changes back into kinetic energy, providing power for the model to enter the ballistic flight phase [48].

**Figure 14:**
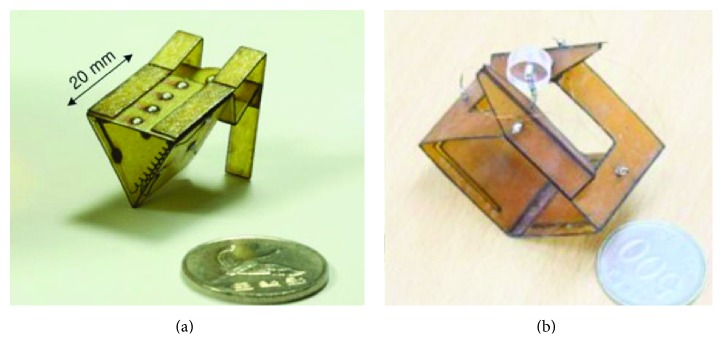
Two prototypes of flea-inspired jumping mechanism. (a) First generation prototype [2]; (b) second generation prototype [50].

The use of new materials also can make robots smaller (millimeter scale or micrometer scale). Considering that the flea can overcome the obstacle 200 times their height, biorobotics laboratory of Seoul National University has developed a multigeneration flea-inspired jumping robots. In 2012, the researchers presented a flea-inspired catapult mechanism for miniature jumping robot, and the shape memory alloy (SMA) spring actuators were used to replace conventional actuators (Figure 14(a)). The jumping leg is a four-bar mechanism with only one DOF, and this robot can jump up to 30 times its body size [2]. On the basis, the researchers also studied the storage capacity of the jumping leg and jumping performance with different stiffness of the leg [49]. In order to achieve active energy storage and active energy release, the research team designed a new flea-inspired jumping mechanism in 2013 (Figure 14(b)). The jumping leg is also a single four-bar linkage with shape memory alloy (SMA) coil spring actuators. The main structure difference between the two mechanisms is that the modified one does not have the coxa, or the body for attaching the actuators [50].

Based on the jumping structure shown in Figure 4(b), biorobotics laboratory of Seoul National University developed many new bioinspired jumping robots, and the movement performance was analyzed in detail. In 2013, a jumping robotic insect was designed, which is shown in Figure 5(a). Laser-cut sheet SMA actuator is employed as the extensor actuator, and its greatest feature is that it can realize active energy storage rather than passive energy storage. The jumping process is shown in Figure 5(b). The robot prototype can achieve jumps of approximately 30 cm with a 2.7 m/s initial velocity. It is 150 times its body height. The air resistance efficiency (jumping height in air (hv)/jumping height in vacuum (hv)) is computed to be 0.83, and the robot exhibits a drag coefficient of 1.8 [51]. In 2014, an insect size micro jumping robot that is inspired by the small jumping insect flea was designed according to the same design principle (Figures 5(c) and 5(d)). The flea-inspired catapult mechanism is the torque reversal mechanism that the stored energy exploded when the force direction of the muscle shifts to the opposite direction with respect to the joint. The jumping height is improved into 40 cm by increasing stored energy compared to the previous prototype shown in Figure 5(a) [52].

**Figure 16:**
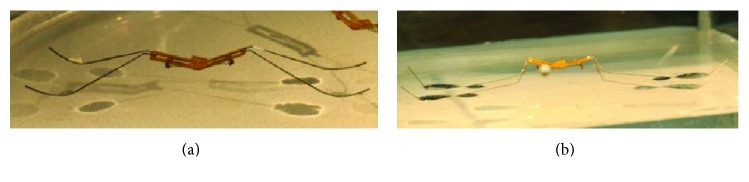
Biowater strider jumping robot. (a) Round shape leg; (b) square shape leg [53].

Similarly, the bioinspired jumping robot with new material also can jump on the water. Biorobotics laboratory of Seoul National University designed a bionic jumping robot which can jump on water by mimicking the jumping movement of water strider. The jumping performance of two kinds of leg structures (a round shape leg and a square shape leg, Figure 16) is analyzed in detail [53]. Compared with the jumping robot which can jump on water shown in Figure 12, this robot has a smaller size and lighter weight.

With the development of bionic jumping robot, the focus of the study is not only on how to achieve rapid takeoff. The researchers gradually made further research on the jumping mechanism, including how to change the takeoff direction, how to improve vertical jumping agility, and how to achieve stable takeoff in complex terrain.

**Figure 17:**
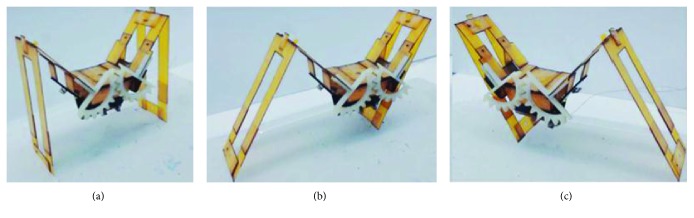
Froghopper-inspired direction-changing mechanism. (a) Initial positions for an upward jump; (b) initial positions for a rightward jump; (c) initial positions for a leftward jump [54].

Takeoff direction control is one of the difficulties in the research of jumping robot. In order to make the jumping robot reach a particular target by controlling their jumping direction, Jung et al. designed a jumping robot by imitating the jumping movement of a froghopper, which can jump by a pair of symmetrically positioned legs and conventional gears. Each leg of this jumping robot has its own thrusting energy, and shape memory alloy (SMA) coil spring actuator is also used. Three jumping postures (synchronous, asynchronous, and single-legged, Figure 17) are tested to investigate how synchronization and moment cancelling affect jumping performance. The results show that synchronous jumping allows the mechanism to change direction from −40° to 40° [54].

In order to improve the vertical jumping agility, Haldane et al. designed a jumping robot Salto to imitate the vertical jumping agility of galago. The vertical jumping agility of the existing jumping robot is up to 55% of a galago. Through use of a specialized leg mechanism, the jumping robot Salto can achieve 78% of the vertical jumping agility of a galago. This robot can jump from the floor to a wall and then springs off the wall to reach a net height that is greater than that accessible by a single jump, and the research results show that series-elastic power modulation is an actuation strategy that enables a clade of vertically agile robots [55].

When the ground is rough or the robot is subject to external interference, the takeoff process may be unstable. At present, there is little research on the takeoff stability of jumping robot, and most of the researches are focused on the jumping stability of the creature. For example, Cofer et al. studied the takeoff stability of locusts. The research results show that the locust can adjust its body posture before takeoff to make the center of gravity be as close as possible to the position of the bouncing force, which is helpful to its steady takeoff [56]. Sutton et al. studied the azimuth control in jumping of froghopper insects. The results show that the initial orientation of the hindleg tibia and the asynchronous movement plays a decisive role in the takeoff direction and roll posture, and the froghopper has easier instability when it jumps with a single leg [57]. For the takeoff stability of robot, Zhang et al. studied the effects of different initial position and posture of a bilateral jumping leg of the bioinspired locust jumping robot on the body posture when the robot jumps on the rough road, which provides a reference for the structural design and control of robots [13].

The typical bioinspired jumping robot with different driving modes is introduced in above paper, and many robots have their own contributions. For Mowgli [30] and the kangaroo jumping robot designed by FESTO [31], design and control of pneumatic system to achieve stable takeoff and landing are the focus of the research. For robots with continuous jumping capability, efficient control methods are proposed to achieve stable jump [14, 36, 38, 48]. For the jumping robot designed by NASA [32], Grillo III [43] and the bionic locust jumping robot designed by Southeast University [45], the diversity of movement function is the main contribution of research. For micro jumping robots, the application of new materials is the biggest difference with the traditional structures of jumping legs [2, 50–52], and this provides a new idea for the design of rigid-flexible coupling bionic structures. The biggest contribution of the fabricated water-jumping robot [47] and biowater strider jumping robot [53] is to achieve a steady takeoff on the water surface. All the above researches are to finally make the jumping robots have good jumping performance, which includes jumping height (obstacle avoidance ability) and jumping distance (escape distance). The difference among them is shown in Table 1.

According to the above quantitative analysis and comparison, existing bioinspired jumping robots can jump with different drives. (1) Pneumatic drive has larger driving force compared with spring drive and flexible material drive, which can drive heavy robots to jump. However, the pneumatic drive has the disadvantages of slower energy release, larger structural size, and complex control systems. Therefore, the bioinspired jumping robot with pneumatic drive has smaller jump height/body height ratio. The pneumatic drive is suitable for large bioinspired jumping robots, and it does not apply to micro robots or the robots with higher requirements for jump height/body height ratio; (2) spring drive is one of the most widely used driving modes. The springs (linear springs or torsion springs) are used to achieve energy storage and rapid release, so that the robot can achieve rapid takeoff no matter if it is a continuous jumping robot or an intermittent jumping robot. Because the size range of spring changes greatly, it can drive both large jumping robots and miniature jumping robots. However, the spring is still a conventional component. When the structure size of the jumping robot is smaller (e.g., the size of the jumping robot is at the micron scale), the spring drive is no longer applicable; (3) the application of new materials can make the bioinspired robots simpler, lighter, smaller, and have better jumping performance. However, the driving force has yet to be further improved. In addition, the relationship between elastic materials and biological structures of creatures remains to be studied.

### 2.2. Air Posture Adjustment Stage

When the posture of the creature with jumping ability is unstable in jumping process, most of them can change their body posture in air to achieve a smooth landing. So the research on the posture adjustment mechanism has always been a focus of attention of researchers. Creatures can adjust body posture in several ways: (1) They adjust air posture by the power generated by air. This method is mainly for creatures with wings. For example, birds or insect can get different lift by the different flapping frequency and amplitude of the bilateral wings to achieve posture adjustment from an aerodynamic point of view [58–62]. (2) They adjust air posture by the swing of other parts of the body, such as spines [63], tail [64, 65], and abdomen [66]. For the posture adjustment of wingless creatures, the earliest study about the body posture was the study of cat falling from high altitude, and the research results show that the spinal bend of the cat in various directions when it falls has an impact on body posture [63]. Since then, many researchers have studied the posture adjustment of the cat [67–69]. The house lizard and lizards, which have long tail, can regulate the body's posture in the fall by swinging its tail [64, 65]. In addition, some creatures can adjust the body posture through the interaction of multiple structures. For example, locust can adjust body posture by its wing, abdomen, and leg [70–72]. According to the posture adjust mechanism of creatures, many posture adjustment structures of the bioinspired jumping robots are designed to achieve stable air posture.

Because wings can make robots glide and it is easier for bioinspired jumping robots to maintain a steady air posture than other structures, the wings are designed for many bioinspired jumping robots. For example, the wing is designed for the bioinspired jumping robot Grillo III in order to extend the hang time, increase the jump distance, and improve the flight stability (Figure 8) [43]. However, the wings of Grillo III are fixed, and the air posture adjustment is passive.

**Figure 18:**
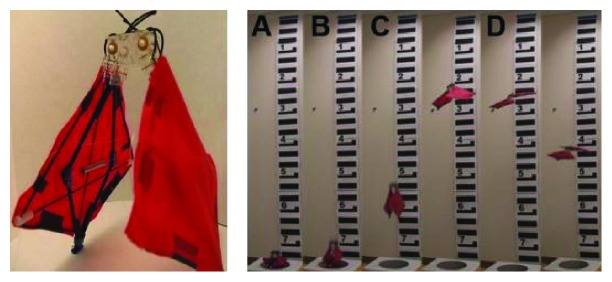
Jumping and gliding robot [73].

In order to make the structure of the jumping robot simpler and the wings be nonfixed structures to meet the needs of the different jumping stage, Woodward et al. designed a micro robot with jumping and gliding integration (Figure 18) inspired from bats which can use the front wing to achieve jumping and gliding. The main structure of the system is bilaterally symmetric and contains two four-bar link mechanisms, and the jumping mechanism and the glider share the same set of mechanisms. After takeoff, the bilateral four-bar link mechanisms unfold and the jumping legs are converted to gliding configuration with semiactive mode. The weigh is about 100 g, and jumping height is 6 m. It can achieve a stable gliding movement [73].

**Figure 19:**
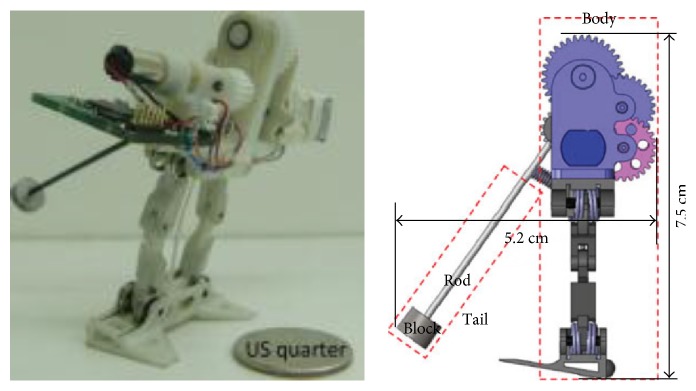
Miniature-tailed jumping robot [74].

Because the tail also has an impact on the air posture, the tail structure is designed for some jumping robots. For the bioinspired jumping kangaroo robot designed by National Taiwan University, an active tail is designed, which has one DOF and can swing under the drive of the motor to adjust the posture of the robot in the air (Figure 13). Three different modes of tail, which include no movement (i.e., stationary tail), movement without sensory input (i.e., fixed tail trajectory, open-loop mode), and movement with body pitch sensory input (i.e., closed-loop mode), are analyzed and compared [48]. Zhao et al. designed a miniature-tailed jumping robot (Figure 19), and it can control its body angle using an active tail to dynamically maneuver in midair for safe landings. The tail also has only one DOF to control the body's pitch angle. The tail and the body are connected by a revolute joint actuated by a DC motor. A motor gear is directly attached to the shaft of the motor, and a tail gear meshes with the motor gear [74]. The experimental results of above two jumping robots show that the swinging of the tail can improve the air posture of the robot effectively.

**Figure 20:**
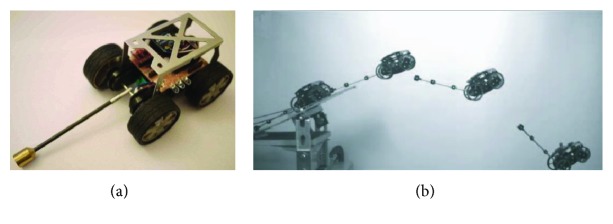
Robot with a one-DOF tail. (a) Robot prototype; (b) posture adjustment process [75].

**Figure 21:**
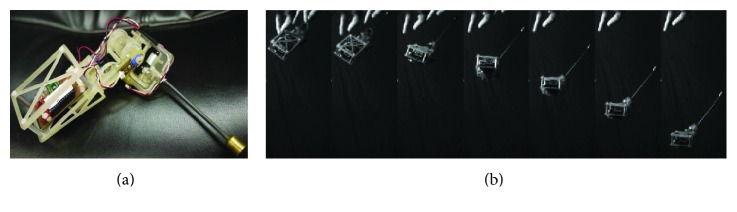
Robot with a two-DOF tail. (a) Robot prototype; (b) Posture adjustment process [76].

Considering lizards can control the swing of their tails to redirect angular momentum from their bodies to their tails, stabilizing body posture in the sagittal plane, Libby et al. designed a lizard-sized robot with an active tail, which can swing up and down in a plane (Figure 20). The research result shows that the tail swung upwards as the controller applied torque to stabilize the body, keeping the body angle constant with PD feedback control [75]. However, the tail can only swing with one DOF. Subsequently, the team improved the tail structure and designed a robot with a two-DOF tail (Figure 21). The robot can achieve very good posture adjustment effect by nonlinear feedback control [76].

**Figure 22:**
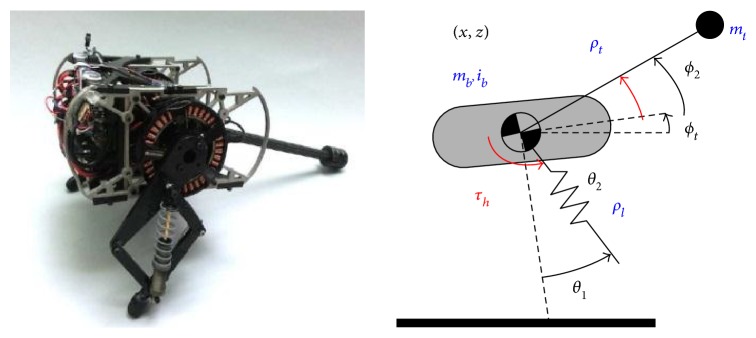
Jerboa robot with a two-DOF tail [77].

The tail of the jerboa robot designed by De et al. also has two DOF. The tail configured as a spherical joint with a point mass at the distal tip. The joint itself is constructed using a linkage such that identical motor displacements result in a pitching motion, and differential motor displacements result in a yawing motion (Figure 22). The hybrid dynamic model is established, and it provides a reference for the tail control [77].

**Figure 23:**
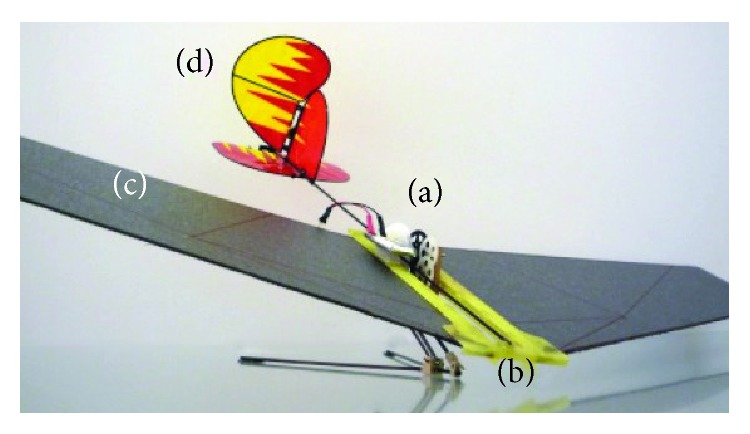
EPFL jumpglider [78].

**Figure 24:**
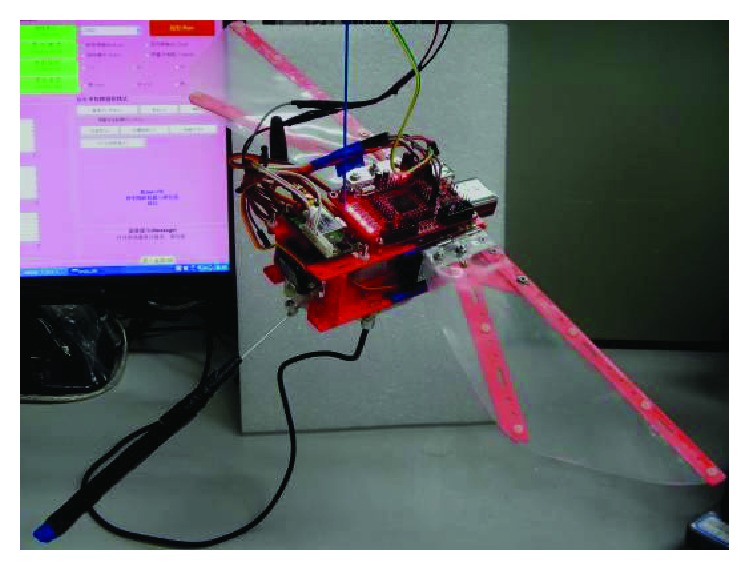
Locust air-posture righting robot [80].

In addition, the combination of various posture adjustment modes is also one of the research emphases. Inspired by the insect wings, Kovac et al. installed the gliding wing on its developed small grasshopper jumping robot to achieve jumping and gliding motion (Figure 23). The wing can keep an upright position after landing for the next takeoff. The tail and rudder system also are designed to change the movement direction and maintain a good air posture. The results from jumpgliding experiments suggest that jumpgliding with rigid wings is the preferable option compared to jumpgliding using a wing-folding mechanism. It increases the jumpgliding distance and reduces the impact energy that has to be absorbed by the robot structure on landing [3, 78, 79]. Chen et al. designed a prototype inspired by the dynamic mechanism of attitude adjustment of locusts (Figure 24). The prototype consists of a pair of wings driven by a four-bar mechanism and a 2-DOF tail to imitate the movement of the locust abdomen. Results show that the pitch and yaw of the tail, and the asymmetric action of the flapping wings, significantly influence the posture of the prototype [80].

The difference of typical air posture adjust method of the bioinspired jumping robot is shown in Table 2.

For the air posture adjustment stage, the studies focus on the structural design for wing and tail and analysis of adjustment mechanism of bioinspired jumping robot. The wings of a jumping robot are mostly fixed wings, and a steady landing can be achieved by gliding. The tails also develop from a one-DOF mechanism toward a multi-DOF mechanism to achieve more complex tuning strategies. However, there is still a big gap between the air posture adjustment mechanism of the robot and that of the creature. There is less research on the air posture adjustment of a robot by flapping wings, real-time control method, and combined action of wing, tail, and abdomen.

### 2.3. Landing Buffering Stage

In nature, many creatures can achieve a stable landing buffer through real-time control of body postures and muscle forces when they fall from high places. Many researchers have studied the landing buffer mechanism of creatures, and toad [81], cat [82–84], dove [85–87], squirrel [88], and bees [89] are the objects of study. For example, the anticipatory hindlimb flexion of toad during the aerial phase is a critical feature for mechanically stable landing [81]. The cat can store kinetic energy and potential energy as a bending deformation energy on the back when landing, thereby reducing the energy absorbed by the limbs [82, 83]. These results have guiding significance for the design of buffering mechanism for bioinspired jumping robots.

**Figure 25:**
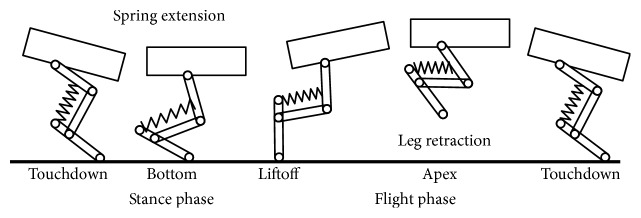
Leg operation of *KenKen* during one stride of hopping [36].

For some bioinspired jumping robot, the jumping legs and the landing legs are the same one. The research focuses on energy storage during landing buffer and energy release for the next jump, and most of this type of jumping robots can achieve good landing buffer. For the jumping robot *KenKen*, leg spring can absorb large impulse at touchdown and transfer its kinetic energy to potential energy for the next stride. Extending the knee yields an extra displacement of the spring, and hence it adds potential energy to the spring. The energy storage of the spring avoids the rigid collision between the leg structure and the ground and achieves better landing buffer. The jumping process is shown in Figure 25 [36].

For the bioinspired jumping kangaroo robot [48], the C-shape structure is used to achieve buffer, which can store energy through the deformation of materials when it lands on the rugged road. Thus, the robot can achieve takeoff and store and release energy in place of the spring. Compared with buffering mechanism with springs being used, C-shape structure has the advantages of simple structure and good buffering performance. Besides, the C-shape structure also can realize the rigidity adjustable by structure design [90].

**Figure 26:**
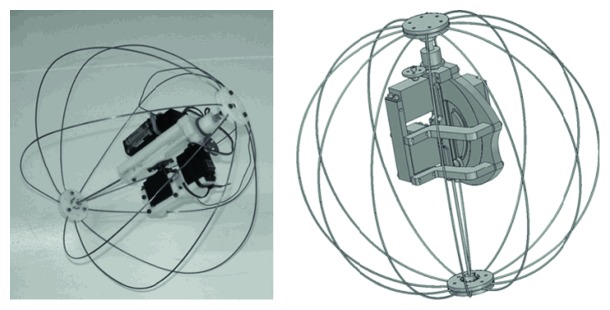
Jumping robot Jollbot [91].

Considering the jump height may be high and the landing posture is uncontrollable, the metal semicircular hoops are designed for some jumping robots (Figure 26). The metal hoop springs allow energy to be stored in a stable material which can drive the robot to jump, and it also makes the robot avoid rigid collision with the ground when the robot lands [91].

**Figure 27:**
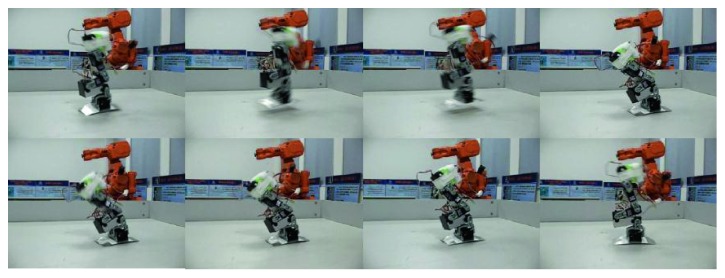
Stable jump process [94].

In addition to the mechanism design, some scholars have further analyzed the landing buffering performance of the bioinspired jumping robot with continuous jumping ability. Wenjie et al. established double mass spring model for bioinspired kangaroo robot, and dynamic stability region and expressions for stability margin of front and rear boundary of the jumping robot are obtained. Then, the relationship between the spring stiffness, touchdown angle, touchdown speed, and landing stability is given, which provides a reference for the robot to achieve a good landing buffer [92]. For the landing buffering process of a one-legged jumping robot, Li et al. studied the orientation stability of planar robot's specific pose in landing phase by establishment of landing equivalent model. The landing stability condition of the one-legged jumping robot is obtained, and the research results provide the design criteria for the planning of the landing phase [93]. During landing impact phase, there is a large impact force. Xu proposed ZMP manipulability to plan jumping gaits based on actuator constraints limitation. The robot can achieve stable movement according to the predesigned stable trajectory under the impact force according to the ZMP plane acceleration orthogonal mapping control strategy. Stable jump process of the robot is shown in Figure 27 [94].

**Figure 28:**
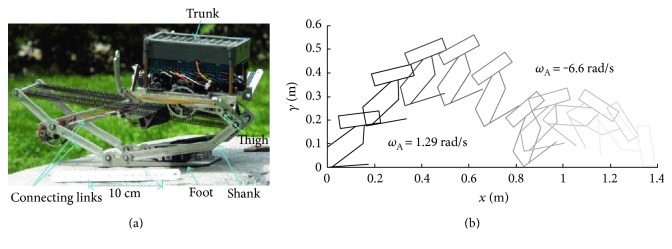
Biokangaroo jumping robot and jump sequence. (a) Prototype of the jumping robot; (b) landing stance of the robot [95].

For intermittent bioinspired jumping robots, Bai et al. designed a biokangaroo jumping robot and landing impact is analyzed. Based on the jump dynamic model and influence rules for landing impact, a technical design of solution is proposed for adjusting the robot's attitude during the jumping and for absorbing the impact energy during the landing. The prototype of the robot and landing process are shown in Figure 28 [95].

If the jumping legs are not exactly the same as the buffering legs for intermittent jumping robot, the buffering leg should be further designed. For example, for the locust-like jumping robot designed by Nguyen et al., the steel wire ring attached at the front plays the role of an absorber that stores the impact energy when the robot crashes to the ground after jumping [44]. For the jumping robot Grillo III, the passive forelegs, which are spring structures, are designed to avoid a rigid collision between the robot and the ground [43]. The above structures are relatively simple. In order to achieve better landing buffer, more complex buffer structure is designed.

**Figure 29:**
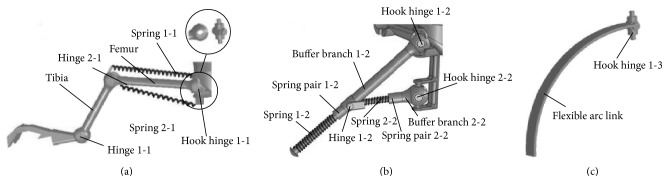
Three leg structure models of buffering legs. (a) Structure of a bionic buffering leg; (b) structure of a multiconstraint buffering leg; (c) structure of an arc buffering leg [34].

Considering the lack of understanding of the differences in the performance of the buffer legs, Zhang et al. analyzed and compared the performance of a bionic buffering leg, multiconstraint buffering leg, and arc buffering leg (Figure 29). The results show that the multiconstrained buffering leg has stronger energy storage capacity; the performance of the bionic buffering leg is poor, but the motion flexibility is higher; the arc buffering leg is simple in structure, and the buffering performance is between the bionic buffering leg and the multiconstrained buffering leg. It provides a reference for the selection of the buffering legs or the structural design of the new type of buffering legs [34].

**Figure 30:**
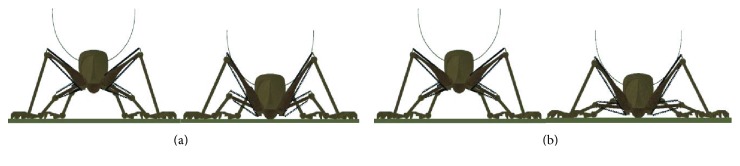
Two buffering processes of a locust. (a) Buffering process with ends of legs fixed; (b) buffering process with ends of legs sliding [17].

Besides, Zhang et al. analyzed two typical buffering methods of locusts, which include the end of the leg fixed and the end of the leg sliding (Figure 30). The dynamic models for different buffering modes are established by Newton-Euler method, and an equivalent nonlinear spring system was established to supplement the buffering process. The buffering performances with different structural dimensions are studied in detail by multiobjective optimization of structural parameters. The results show that the mechanism with the ends of the legs sliding can obtain a better dynamic performance, which is consistent with the experimental observation results [17, 96].

**Figure 31:**
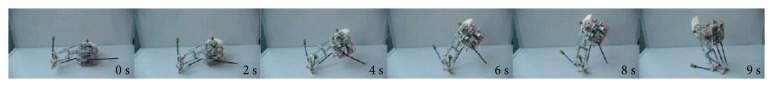
Self-righting process of a jumping robot designed by Zhang et al. [16].

**Figure 32:**
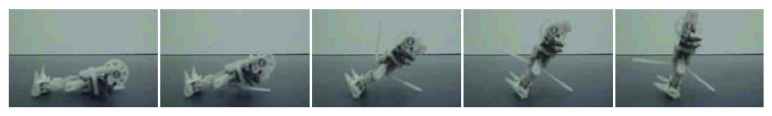
Self-righting process of a jumping robot designed by Zhao et al. [99].

In particular, the jumping robots are easy to rollover or roll forward after landing and further lead to a series of problems [97]. Considering the insects with jumping ability can adjust the body posture after rolling over [98], so for bioinspired jumping robots, the recovery of body posture is an important performance that robots should have. For example, the self-righting mechanism is designed for a bioinspired jumping robot designed by Zhang et al. [16]. The self-righting process is composed of two stages: actively propping (AP) and passively self-righting (PS). The AP stage is the process when the robot uses the pole leg to prop its body up step by step. In the PS stage, the robot will passively self-right under its gravity and rotational inertia (Figure 31). Zhao et al. design a self-righting mechanism for a controllable and continuous jumping robot. The two legs of self-righting mechanism can rotate with respect to the body in opposite directions simultaneously. In this way, the robot can stand up with either of the two large surfaces touching the ground (Figure 32) [99]. In this way, the bioinspired jumping robots can achieve steady jumps.

The research on the buffering process of the bioinspired jumping robot mainly involves the design of buffering structure, the analysis of buffering performance, and the posture adjustment after overturning. Though the energy absorption of the jumping robots during landing is achieved and the rigid collisions are avoided, the existing jumping robots, especially for the intermittent jumping robots, are still unable to achieve a good and stable landing buffer in complex environments. The relationship among takeoff performance, joints attitude of buffering structures, and landing buffering performance (including mechanical performance and complex environmental adaptability) of jumping robots needs to be further analyzed and verified by experiments.

At present, the research on bioinspired jumping robots has made a series of achievements, showing a wide application prospect. According to the above research status, the bioinspired jumping robots are developing from macroscopic bionics study to miniaturization and integration of material and structure, and the structures and motion functions are getting closer to the creatures. Researchers have made more study on takeoff of bioinspired jumping robot, and a great deal of research results is obtained. The researches on air posture adjustment and landing buffering start relatively late but also achieved some results in structural design and performance analysis.

## 3. Discussion

It can be seen from the existing research that there is still a big difference for the motion ability between bioinspired jumping robots and creatures. For example, the existing bioinspired jumping robots cannot achieve the air posture adjustment in a short time like creatures under the combined action of wings, abdomen, and tail. The study of bioinspired jumping robots still has the following limitations.

1. The study of biological mechanism is not thorough, and the equivalent model is simple. The key principles and functional characteristics of the creatures should be revealed by the study of biological mechanism to provide the basis for the research of bioinspired jumping robots. The key to the study is how to accurately analyze and model the biological movement mechanism. Although many researchers have studied the movement mechanism of creatures by the combination of theory and experiment, the research results still cannot fully reveal the physiological characteristics of the creatures. For example, the dynamic model of hindlegs of adult desert locusts is established by Omer et al. to analyze the jumping mechanism and takeoff trajectory [26]. But only the macroscopic motion characteristics of hindlegs are analyzed, and muscle movement patterns and movement efficiency are still not considered. This leads to the inability to effectively design bionic muscles to achieve jumping.
  The above limitations are caused by the following reasons. First of all, the creature is a very complex system, and each movement function (i.e., takeoff, air posture adjustment, and landing buffer) is influenced by many factors, such as bone, muscle, and nervous system. Therefore, it is very difficult to establish a precise and complete model. Secondly, the study of biological mechanism involves multidisciplinary intersection. For example, the research on jumping ability of locusts involves biology, biomechanics, and mathematics. The research methods of interdisciplinary need to be further improved.
  Therefore, the lack of research on biological movement mechanism is one of the important factors that restrict the development of bioinspired jumping robots.
2. The design method of bionic structure of the bioinspired jumping robot is far from the rationality and ingenuity of biological structure, and the motion function is single. The key principles of creatures should be applied to the structure design of the robot, and thus the robot can simulate the movement function of the creatures. However, both the structure and the driving mode of the jumping robots are not able to reflect the key characteristics of creatures.
  The creatures have rigid-flexible coupling structure, which can enhance its movement performance and environment adaptability. However, the structures of most of the existing bioinspired jumping robots are rigid and the soft tissue structures are not well considered. For example, the leg muscles of locust have been studied [19], but the traditional spring structures are used for most jumping robots to replace the muscles, which is different from the movement mechanism of muscles. And the equivalent relationship between the springs of the robots and the muscles of creatures is not studied in depth, which makes the design and installation of spring lack of basis [44–46]. Although flexible structures are applied for some robots [2, 49–54], the difference between the movement performance of the flexible structures and biological movement mechanism is not analyzed in detail. In addition, most of the robots do not pay much attention to the microscopic characteristics and structural optimization analysis, and the optimization process is too simplified. For example, parameter optimization of one-DOF jumping legs was conducted by Zhang et al. to make the motion law of the jumping leg closer to that of the hindleg of a locust [13]. However, the optimization target is only the end trajectory of jumping leg, and the scale effect and other performances are not considered.
  The drive modes of creatures have the characteristics of small and simple structures, large driving force, and high drive efficiency. However, regardless of the pneumatic drive, spring drive, or flexible material drive, they cannot meet the above advantages at the same time and do not reproduce the key features of the creature. Therefore, the driving mechanism of the jumping robot needs further study.
  In addition, the bioinspired jumping robot can not only realize fast takeoff but also should have other movement ability, such as air posture adjustment, landing buffering, and crawling, so as to meet the needs of practical application. However, the existing structures cannot meet the needs of multiple motion functions.
3. The research of new bionic materials with high performance is insufficient, and the existing research results have not been effectively applied. Bionic material has the most reasonable macroscopic and microscopic structure and has the ability of self-adaptation and self-healing [100]. Therefore, the application of bionic materials can help bioinspired jumping robots to reproduce key functional features of creatures, such as biological drag reduction [101, 102], wear resistance [103], fatigue resistance, and self-cleaning [4]. At present, the research of bionic materials, which can take the place of leg muscles and tendons, should be further studied in the engineering application, model establishment, and preparation methods [104]. For example, the tarsus of locust has flexible characteristic [105, 106], and it has an impact on landing buffer. However, the bionic tarsus material still has not been applied to locust-like jumping robots.
4. The bionic control method is traditional, and the study of new bionic control method is not enough. For most of the existing jumping robots, the drive is used as a jumping trigger device. So the robot is almost uncontrolled after the takeoff phase (the leg posture of some jumping robot can be changed in the air phase, but it is adjusted passively [36]). This control method differs greatly from the real-time control of creatures. Besides, some problems, such as adaptive (adaptability of complex environment for jumping robots), group control (movement control of multiple jumping robots), and class evolution (self-learning ability of jumping robots), also need to be studied through the further research of control methods. In addition, good environment perception ability is one of the research directions of bioinspired jumping robots. The existing jumping robots cannot simulate the perceptual characteristics of the creature accurately, and it restricts the application of bioinspired jumping robots in complex environments.
5. The energy conversion efficiency is low. The jumping process of robots is the process of energy release and absorption. Energy utilization of creatures is very high after long natural evolution. The energy conversion efficiency of creatures is as high as 100%, and the conversion efficiency of the chemical energy from muscle to mechanical energy is close to 50%, which is far more than that of all kinds of jumping robots [107]. However, there is no detailed study on the energy utilization and consumption of bioinspired jumping robots based on bioenergy conversion mode.

In a word, key principles of creatures have not been well revealed, and they are not well applied to the design of bioinspired jumping robots. Although some scholars have carried out targeted research for bioinspired jumping robots from the point of view of critical jumping performance [55], the jumping performance of the robot still needs to be improved. The study of bioinspired jumping robots should combine the new theories and methods of modern mechanics and robotics with complex biological macroscopic and microscopic characteristics to realize the unity of structural bionic, material bionic, functional bionic, and controlled bionic researches to make the key principles of creatures applied to the jumping robots [108]. The development trend of bioinspired jumping robots is as follows:

1. With the application of biology, chemistry, physics, mechanics, and other disciplines in the study of the bionic mechanism, the research of bionic mechanism will develop from macroscopic to microcosmic. The equivalent model will be more accurate, which can provide a theoretical basis for the design of bioinspired jumping robots.
2. Bionic rigid-flexible coupling structures, which contain macrostructure (i.e., wings and legs) and microstructure (i.e., soft tissue), will be applied to the design of bioinspired jumping robot, which makes the robot not only with a rigid support structures but also with a flexible adaptive structures. Besides, a jumping robot can achieve even more complex movements, such as takeoff, air posture adjustment, landing buffer, and crawl.
3. Bionic materials which are closer to biological properties will be used to obtain the advantages of low energy consumption, high efficiency, and strong environmental adaptability. In particular, bionic materials can be applied not only to macrostructures [2, 49, 50] but also to microstructures [109].
4. The traditional triggered control methods for most jumping robots will be abandoned, and the research focuses on the real-time control [110] and electronic nervous systems [111] to achieve more accurate movement. For example, the postures of the jumping legs and buffering legs will be adjusted in real time according to the terrain characteristics. Besides, multisensory information fusion, remote monitoring, and coordinated control of multiple motion functions will be studied to achieve more accurate and better adaptable control process and better environment perception ability.
5. Energy conversion process is converted from inefficient mechanical energy to efficient biological energy. The research results about conversion mechanism of biological energy will be applied to the study of energy conversion process of bioinspired jumping robots. The research focuses on the transmission efficiency, energy loss, and the relationship between functional requirements and energy transmission mode, so as to improve the energy efficiency and reduce energy consumption of the bioinspired jumping robots.

## 4. Conclusion

Bioinspired jumping robots can overcome high obstacles by simulating motion process of creatures with jumping ability and have good adaptability to complex environment, so it has a wide application prospect. On the basis of revealing physiological structure and motor function of creatures, the researchers study the bioinspired jumping robots based on the three stages of the jumping process, which includes takeoff stage, air posture adjustment stage, and landing buffering stage. However, the functional features of the existing bioinspired jumping robots still differ greatly from the creatures that are imitated. This is because there are still some shortages in the research of jumping robots, such as the research of bionic mechanism is not thorough and the structure, material, control method, and energy conversion mode are different from key principles of creatures, which limits the development of bioinspired jumping robots. In the future, the research results of bionic mechanism will be applied to the design of bioinspired jumping robots, so that a jumping robot can be applied in practice.

## Figures and Tables

**Figure 15:**
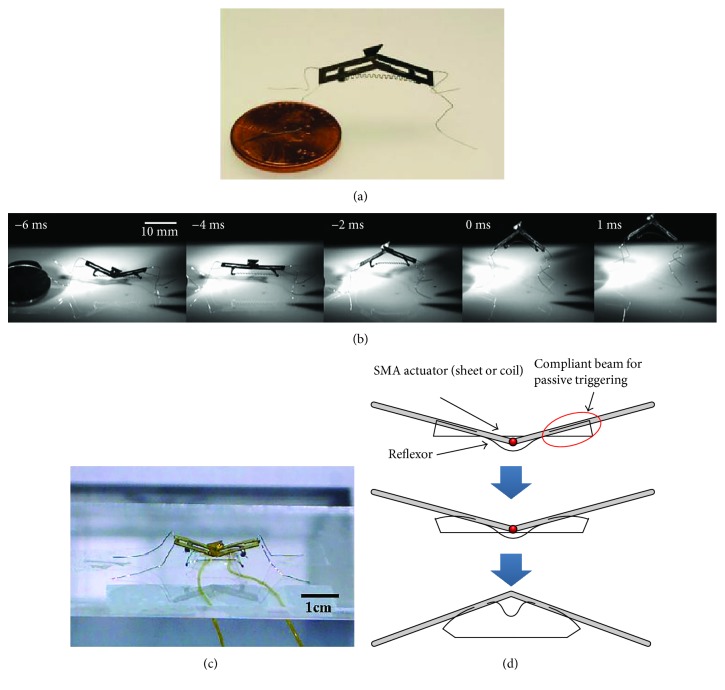
Insect size micro jumping robot with bilateral jumping leg structure. (a) The prototype of jumping robot [51]; (b) takeoff process of jumping robot [51]; (c) prototype of the micro jumping robot [52]; (d) the triggering procedure of the flea-inspired catapult mechanism [52].

**Table 1:** Comparison of takeoff performance of different bioinspired jumping robots.

Driving mode	Robot	Leg mechanism	Weight/kg	Jumping height/m	Jumping distance/m	Jump height/body height ratio
Pneumatic drive	Mowgli [30] (University of Tokyo)	Bionic serial leg	3	0.5	—	More than 0.5
	Kangaroo jumping robot [31] (FESTO)	Four-bar mechanism	7	0.4	0.8	0.67

Spring drive	Jumping robot [32] (NASA)	Six-bar mechanism	1.3	0.8	1.8	5.33
	Uniroo [14] (MIT)	Bionic serial leg	6.6	0.1	—	0.16
	KenKen [36] (University of Tokyo)	Bionic serial leg	13.26	0.55	—	0.65
	Chobino1D [38] (Vrije Universiteit Brussel)	Bionic serial leg	—	0.4	—	1
	Grillo [40] (IMT Lucca Institute for Advanced Studies)	Similar-bionic leg	20 × 10^−3^	0.15	—	5
	Grillo III [43] (Zhejiang University)	Four-bar mechanism (similar-bionic)	22 × 10^−3^	0.2	0.1	8
	Jumping robot [44] (Konkuk University)	Similar-bionic leg	7 × 10^−3^	0.71	1	14
	Jumping robot [45] (Southeast University)	Four-bar mechanism	154 × 10^−3^	0.99	0.73	8.26
	Jumping robot [46] (Ort Braude College)	Bionic serial leg	23 × 10^−3^	3.35	1.37	74.4
	Fabricated water-jumping robot [47]	Similar-bionic leg	10.2 × 10^−3^	0.12	0.41	—

Flexible material drive	Bionic kangaroo robot [48] (National Taiwan University)	Half-circular leg	10.8	0.2	0.3	1.1
	Flea-inspired jumping mechanism (first generation) [2]	Four-bar mechanism (posterior, SMA)	1.11 × 10^−3^	0.64	0.35	30
	Flea-inspired jumping mechanism (second generation) [50]	Four-bar mechanism (bilateral, SMA)	2.25 × 10^−3^	1.2	—	40
	Jumping robotic insect [51] (Seoul National University)	Two links mechanism (SMA)	34 × 10^−6^	0.3	—	150
	Micro jumping robot [52] (Seoul National University)	Two links mechanism (SMA)	36 × 10^−6^	0.4	—	200
	Biowater strider jumping robot (Harvard University) [53]	Two links mechanism (SMA)	68 × 10^−6^	0.142	—	—

**Table 2:** Comparison of air posture adjustment methods of different bioinspired jumping robots.

Robot	Posture adjustment mechanism	Adjustment mode	Movement mode of posture adjustment mechanism
Grillo III [43] (Zhejiang University)	Wing	Passive adjustment	Fixed
Jumping and gliding robot [73] (Carnegie Mellon University)	Wing	Passive adjustment	Semiactive transition
Bionic kangaroo robot [48] (National Taiwan University)	Tail	Active adjustment	One-DOF tail (swings up and down)
Miniature-tailed jumping robot [74] (Michigan State University)	Tail	Active adjustment	One-DOF tail (swings up and down)
Lizard-sized robot [75] (University of California, Berkeley)	Tail	Active adjustment	One-DOF tail (swings up and down)
Tailed robot [76] (University of California, Berkeley)	Tail	Active adjustment	Two-DOF tail
Jerboa robot [77] (University of Pennsylvania)	Tail	Active adjustment	Two-DOF tail
EPFL jumpglider [3, 78, 79] (Harvard University)	Wing/tail	Passive adjustment of wing/active adjustment of tail	Wing unfolding/tail movement
Locust air-posture righting robot [80] (Beihang University)	Wing/ tail	Active adjustment of wing/ active adjustment of tail	One-DOF wing/two-DOF tail
